# Associations of serum pepsinogen and Gastrin-17 with *Helicobacter pylori* infection, sex, and age, in an asymptomatic coastal population: A cross-sectional study in Rizhao, China

**DOI:** 10.1371/journal.pone.0335228

**Published:** 2025-11-04

**Authors:** Tianyi Zhang, Xiangxiang Meng, Xiaomei Wang, Shanwen Mi, Yanjie Qu, Jianxin Li, Sujun Hou, Yaocai Wang, Mengmeng Yin

**Affiliations:** 1 Department of Clinical Laboratory, Rizhao Hospital of Traditional Chinese Medicine, Rizhao, China; 2 Department of Spleen and Stomach Diseases, Rizhao Hospital of Traditional Chinese Medicine, Rizhao, China; Tehran University of Medical Sciences, IRAN, ISLAMIC REPUBLIC OF

## Abstract

**Background:**

Pepsinogen (PG) and gastrin-17 (G-17) are widely used in the screening of gastric diseases. Our cross-sectional clinical study investigates the relationship between *Helicobacter pylori* (HP) infection, sex, and age on serum levels of PG and G-17 in asymptomatic subjects in Rizhao, China.

**Methods:**

A total of 12,746 asymptomatic subjects were enrolled in the study between August 2023 and January 2024. Serum levels of pepsinogen I (PGI), pepsinogen II (PGII), and G-17 were measured using the chemiluminescent microparticle immunoassay method, and the PGI/PGII ratio (PGR) was calculated. HP infection was detected using the Colloidal Gold Method, and the relationship between age, sex, HP infection, and serum PG and G-17 levels was analyzed.

**Results:**

HP prevalence was 19.33% in this study. The serum PGI, PGII, and G-17 levels were significantly higher in the HP-positive group compared to the HP-negative group (P < 0.001), whereas the PGR was notably lower in the HP-positive group (P < 0.001). Spearman correlation tests analysis indicated a positive correlation between HP infection and PGI, PGII, and G-17 (r = 0.144, P < 0.001; r = 0.418, P < 0.001; r = 0.268, P < 0.001), and a negative correlation with PGR (r = −0.438, P < 0.001). ROC curve shows that the AUC of the combination of PGI, PGII, PGR, and G-17 in diagnosing HP positive were 0.605 (95% CI: 0.592–0.618), 0.805 (95% CI: 0.795–0.816), 0.820 (95% CI: 0.811–0.830), and 0.709 (95% CI: 0.698–0.720), respectively. The detection rates of abnormal PG and G-17 levels were significantly higher in the HP-positive group than in the HP-negative group (P < 0.01). Males exhibited significantly higher levels of both PGI and PGII than females (P < 0.001). The G-17 levels were higher in males than females in the 50–59 age group (P < 0.05). Spearman correlation analysis revealed that serum levels of both PGI and PGII exhibited an increase with age. Serum PGI, PGII, and G-17 levels were positively correlated with age, although the relationship was weak (r = 0.228, P < 0.001; r = 0.246, P < 0.001; r = 0.042, P < 0.001). Following adjustment for sex and Helicobacter pylori infection covariates using a restricted cubic spline (RCS), the analysis revealed a significant overall association between PGI, PGII, PGR, and G-17 serum levels and age (P for overall < 0.001, P for overall < 0.001, P for overall = 0.003, and P for overall < 0.001, respectively). Furthermore, a nonlinear relationship was found between PGI, PGR, and G-17 levels and age (nonlinear P = 0.004, nonlinear P = 0.001, and nonlinear P = 0.008, respectively), whereas PGII exhibited a linear correlation (nonlinear P = 0.841).

**Conclusion:**

Serum levels of PG and G-17 are associated with HP infection, sex, and age. These findings provide region-specific insights into the relationships between these biomarkers and HP infection, highlighting the importance of considering HP infection status, sex, and age in future research.

## Introduction

Gastric cancer (GC) is a common malignant gastrointestinal tract malignancy. Approximately 40 percent of new cases and deaths worldwide occur in China, ranking the disease as third in terms of mortality [1,2]. Currently, endoscopy combined with histopathological examination is the gold standard for diagnosing GC. However, large-scale endoscopic screening is impracticable for health examinations in China due to its invasiveness and high cost [3,4]. Given these challenges, there is a pressing need to explore cost-effective and less invasive screening alternatives to improve early detection and reduce the burden of gastric cancer in the population.

Serum pepsinogen (PG), the inactive precursor of pepsin, comprises two isoforms: pepsinogen I (PGI) and pepsinogen II (PGII). Its levels reflect the number of glands and cells in the gastric corpus mucosa [5–7]. Dysregulated PG expression and a progressive reduction in the PGI/PGII ratio (PGR) have been reported to play a pivotal role in the progression from normal gastric mucosa to precancerous lesions and, ultimately, to GC [8,9]. Consequently, serum PG serves as a non-invasive biomarker for evaluating the morphological and functional status of the gastric mucosa.

Gastrin 17 (G-17) was another non-invasive biomarker for reflecting the gastric antrum mucosa’s atrophy and pathological changes [10]. G-17, almost exclusively secreted by antral G-cells and recognized as the predominant form of gastrin in plasma or tissue within the antral mucosa, plays a vital role in regulating gastric acid secretion and promoting the growth of the gastric mucosa [11,12]. Some scholars have suggested using G-17 in combination with PG as a serological screening marker to distinguish pathological conditions, such as *Helicobacter pylori* (HP) associated gastritis and atrophic gastritis, from healthy states [13,14].

HP infection plays an important role in the progression of the cascade progression from gastric lesions to GC [15]. Non-invasive biomarkers like serum PG and G-17 are crucial for early detection. However, few studies have examined the characteristics of biomarker levels, including serum PG and G-17, with factors such as age, sex, and HP infection in asymptomatic populations from coastal regions in China. This study analyzed a large cohort of asymptomatic Chinese individuals from Rizhao City, a coastal area in China, to explore the associations between serum PG and G-17 levels and variables, including HP infection status, sex, and age, through rigorous statistical methods. These findings aim to enhance our understanding of early gastric health and provide valuable insights to inform preventive strategies for gastric disease management.

## Materials and methods

### Subjects

This was a cross-sectional study of an asymptomatic population. We enrolled 15,831 asymptomatic subjects living in the area of Rizhao City, Shandong Province, China, between August 2023 and January 2024. The inclusion criteria are asymptomatic subjects, main residence in the local study area, and the ability to give written consent for study procedures. To minimize confounding effects from conditions known to alter serum gastrointestinal biomarkers (PG and G-17) [16–21], we excluded subjects with: (1) subjects with previous surgery, chemotherapy, or radiotherapy; (2) subjects administered acid inhibitors or antibiotics within the past month; (3) subjects with a history of HP eradication; (4) subjects with bleeding tendency or those who have blood coagulation dysfunction; (5) subjects with a history of gastric pathologies, heart, liver, brain, or kidney diseases; (6) incomplete data. This research was conducted at the Rizhao Hospital of Traditional Chinese Medicine. Finally, 12,746 subjects of the total number were enrolled in this study, including 5,962 males (46.78%) and 6,784 females (53.22%), aged 17–98 years (mean, 60.69 ± 10.13). Although no strict age restrictions were imposed, the sample was predominantly composed of middle-aged and elderly individuals.

The written consents were obtained from all subjects. This study was approved by the Ethics Committee of the Rizhao Hospital of Traditional Chinese Medicine and conducted following the 1996 Declaration of Helsinki. (Ethical approval number: AF/SX-08/01.0.2021–024).

### PG and G-17 determination and analysis

Five mL of fasting venous blood was collected from each subject and centrifuged at 3000 r/min for 10 min to obtain the serum. Serum PGI, PGII, and G-17 levels were assayed by the chemiluminescent microparticle immunoassay method in ‌MAGLUMI X8 using the Snibe matching assay kit (Shenzhen New Industries Biomedical Engineering Co., Ltd., China), and the PGI/PGII ratio (PGR) was calculated. The following criteria were used to assess the concentrations according to the threshold values of the assay kit: (1) PGI < 70.0 ng/mL or PGI > 240 mg/L was considered abnormal; (2) PGII > 13.0 ng/mL was considered abnormal; (3) a PGI/PGII ratio (PGR) < 3 was considered abnormal, and (4) G-17 > 7.6 pmol/L was considered abnormal.

The reference ranges for PG and G-17 were established following the Clinical and Laboratory Standards Institute (CLSI) EP28-A3C guideline and the WS/T402-2012 guideline issued by the Ministry of Health of the People’s Republic of China. These reference intervals were derived from serum samples of healthy Chinese adults, with the 2.5th and 97.5th percentiles defining the lower and upper limits, respectively. Rigorous standardization procedures were employed to ensure the scientific validity and reliability of the reference values.

### Anti-HP IgG test

An existing HP infection was performed by HP Antibody (HP-Ab) Assay Kit by Colloidal Gold Method (Maccura Biotechnology Co., Ltd, China). The test involves adding one drop (approximately 35 μL) of serum to the sample well, followed by two drops of diluent, and reading results within 10–15 minutes under natural light, avoiding strong light or shadows that may interfere with interpretation. The positive test criteria were the following: (1) negative (-): Only the control line (C-line) appears, indicating no HP antibodies detected or the antibody concentration is below the detection threshold; (2) positive (+): Both the control line (C-line) and the test line (T-line) appear, indicating the presence of HP antibodies, regardless of the intensity of the test line color; (3) invalid: If the control line (C-line) does not appear, the test is invalid and should be repeated.

### Statistical analysis

Statistical analyses were performed using SPSS 25.0 (IBM SPSS, Chicago, IL, USA). Continuous data were tested for normal distribution using the Kolmogorov-Smirnov test. Data with normal distribution were represented by mean ± standard deviation (SD); data with skewed distribution were presented as median (interquartile range) (IQR). Categorical variables were expressed as (%), and the Chi-square test of independence was used to assess associations between group variables (sex, age categories) and HP infection status. Differences between groups were assessed using the Mann-Whitney U test or the Kruskal-Wallis test. To account for multiple comparisons, the Bonferroni correction was applied to adjust the significance level, ensuring control of the overall false positive rate. The correlation between all PG parameters and G-17 levels, and age was analyzed separately by a bivariate Spearman correlation test. Additionally, restricted cubic spline (RCS) analysis was employed to explore potential nonlinear relationships between these variables and age. To evaluate the diagnostic performance of each biomarker combination, separate binary logistic regression models were constructed using the corresponding biomarker concentrations as predictors. Each model yielded a predicted probability of HP infection for each subject. Receiver operating characteristic (ROC) curves were then generated based on these predicted probabilities, and the optimal probability threshold for classification was determined by maximizing Youden’s Index (sensitivity + specificity – 1). Subjects were classified as HP-positive if their predicted probability exceeded this optimal threshold; otherwise, they were classified as HP-negative. P < 0.05 was considered to indicate a statistically significant difference.

## Results

### Associations of serum PG and G-17 levels with HP infection

Compared to the non-infected group, serum PGI, PGII, and G-17 levels were significantly higher in HP-positive individuals (P < 0.001, P < 0.001, P < 0.001), while PGR was lower (P < 0.001). In addition, compared with the PGI level, the PGII and G-17 levels in subjects who were HP-positive dramatically increased (Table 1). Moreover, Spearman correlation tests analysis indicated a positive correlation between HP infection and PGI, PGII, and G-17 (r = 0.144, P < 0.001; r = 0.418, P < 0.001; r = 0.268, P < 0.001), and a negative correlation with PGR (r = −0.438, P < 0.001) (Table 2).

**Table 1:** HP infection and serum PG and G-17 levels.

	n	PGI (ng/mL)	PGII (ng/mL)	PGR	G-17 (pmol/L)
HP+	2464	117.00 (87.50,161.00)	19.70 (12.90, 29.58)	6.21 (4.72, 8.26)	6.30 (3.73, 13.20)
HP-	10282	97.6 (77.98, 127.00)	9.65 (7.87, 12.50)	10.18 (8.57, 11.88)	3.09 (2.03, 5.74)
Z		−16.283	−47.153	−49.466	−32.259
P		<0.001	<0.001	<0.001	<0.001

HP: *Helicobacter pylori*; PGI: Pepsinogen I; PGII: Pepsinogen II; PGR: PGI/PGII Ratio; G-17: Gastrin-17.

**Table 2:** Spearman Correlation Analysis of HP Infection, Gender, Age with Serum Markers.

		PGI	PGII	PGR	G-17
HP	r/P	0.144/ < 0.001	0.418/ < 0.001	−0.438/ < 0.001	0.286/ < 0.001
sex	r/P	0.177/ < 0.001	0.168/ < 0.001	0.016/0.077	0.011/0.212
age	r/P	0.228/ < 0.001	0.246/ < 0.001	−0.006/0.513	0.042/ < 0.001
HP Positive					
sex	r/P	0.037/0.064	0.082/ < 0.001	−0.076/ < 0.001	0.030/0.142
age	r/P	0.072/ < 0.001	0.185/ < 0.001	−0.170/ < 0.001	0.036/0.071
HP Negative					
sex	r/P	0.219/ < 0.001	0.216/ < 0.001	0.029/0.004	0.010/0.321
age	r/P	0.278/ < 0.001	0.311/ < 0.001	0.010/0.332	0.054/ < 0.001

HP: *Helicobacter pylori*; PGI: Pepsinogen I; PGII: Pepsinogen II; PGR: PGI/PGII Ratio; G-17: Gastrin-17

**Fig 1:**
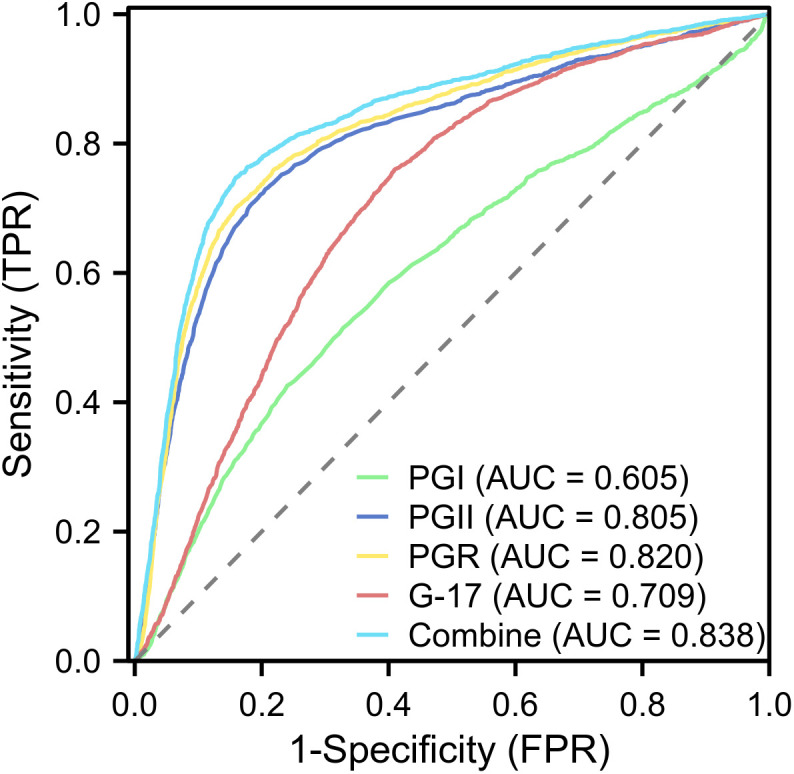
The ROC curves for PGI, PGII, PGR, and the G-17. PGI: Pepsinogen I; PGII: Pepsinogen II; PGR: PGI/PGII Ratio; G-17: Gastrin-17; ROC: receiver-operating characteristic curve.

The AUC values for diagnosing HP-positive cases with PGI, PGII, PGR, and G-17 were 0.605 (95% CI: 0.592–0.618), 0.805 (95% CI: 0.795–0.816), 0.820 (95% CI: 0.811–0.830), and 0.709 (95% CI: 0.698–0.720), respectively (Fig 1). The diagnostic performance of the combined markers (PGI, PGII, PGR, and G-17) showed an AUC of 0.838 (95% CI: 0.828–0.848), with a sensitivity of 74.76% and a specificity of 84.09%, indicating a significantly improved diagnostic efficacy compared to individual markers (S1 Table).

### Relationships of serum PG and G-17 levels with sex

The Spearman correlation analysis revealed weak positive correlations between male and PGI (r = 0.177, P < 0.001) and PGII (r = 0.168, P < 0.001) in the overall cohort, with stronger associations in HP-negative individuals (PGI: r = 0.219, P < 0.001; PGII: r = 0.216, P < 0.001). In HP-positive subjects, however, no significant correlation was observed for PGI (r = 0.037, P = 0.064), while PGII retained a weak positive correlation (r = 0.082, P < 0.001).

**Fig 2:**
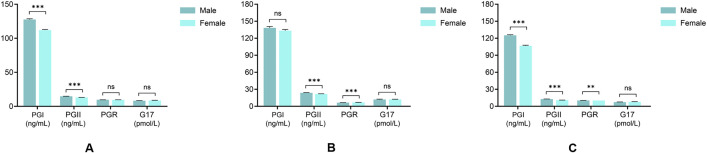
Serum PG and G-17 levels in different sexes. (A) Comparative analysis of serum PGI, PGII, PGR, and G-17 levels by sex in all HP status groups; (B) sex-based disparities in serum PGI, PGII, PGR, and G-17 levels in HP-positive individuals; (C) sex-based disparities in serum PGI, PGII, PGR, and G-17 levels in HP-negative individuals. ***P < 0.001 compared to females. **P < 0.01 compared to females. PGI: Pepsinogen I; PGII: Pepsinogen II; PGR: PGI/PGII Ratio; G-17: Gastrin-17.

Fig 2 presents the analysis of sex-based differences in serum levels of PGI, PGII, PGR, and G-17 across different HP infection statuses. PGI levels were significantly higher in males than females in the overall cohort (P < 0.001) and HP-negative subgroup (P < 0.001), but not in HP-positive individuals. In contrast, PGII levels were elevated in males across all cohorts (P < 0.001 for all groups). For PGR, males had higher values than females in the HP-negative subgroup (P < 0.01), whereas lower values were observed in HP-positive males (P < 0.001). No sex differences in G-17 levels were detected in any group.

### Relationships of serum PG and G-17 levels with age

**Fig 3:**
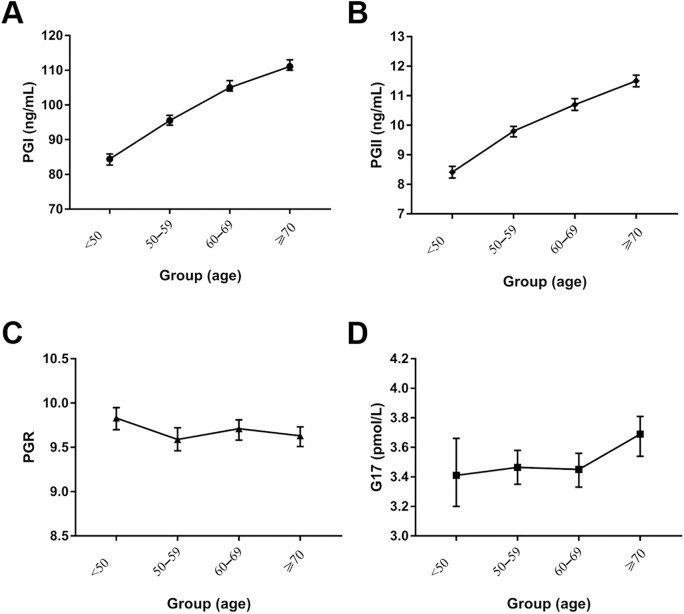
Serum PG and G-17 levels in various age groups. The serum PG and G-17 concentrations were determined in 12,746 cases, which have been divided into 4 groups, including < 50 years-old group (n = 1,730), 50−59 years-old group (n = 3,676), 60−69 years-old group (n = 4,080), > 70years-old group (n = 3,260). PGI: Pepsinogen I; PGII: Pepsinogen II; PGR: PGI/PGII Ratio; G-17: Gastrin-17.

In 12,746 subjects, Serum levels of both PGI and PGII increased with age. As suggested by the Kruskal-Wallis H test of multiple independent samples, the differences in the serum PGI, PGII, PGR, and G-17 levels were statistically significant between various age groups (P < 0.001, P < 0.001, P = 0.003, P < 0.001) (Fig 3, Table 3).

**Table 3:** Comparison of serum PG and G-17 levels in various age groups.

Group (y)	n	PGI (ng/mL)	PGII (ng/mL)	PGR	G-17 (pmol/L)
<50	1730	84.45 (77.20, 125.00)	8.42 (6.77, 11.50)	9.83 (8.13, 11.42)	3.41 (2.08, 7.00)
50–59	3676	95.50 (74.90, 123.00) *	9.80 (7.81, 14.50) *	9.59 (7.48, 11.38) *	3.47 (2.17, 6.68)
60–69	4080	105.00 (83.42, 136.00) *^#^	10.70 (8.60, 15.60) *^#^	9.71 (7.57, 11.60)	3.45 (2.14, 6.74)
≥70	3260	111.12 (85.73, 152.00) *^#$^	11.50 (9.10, 17.40) *^#$^	9.63 (7.37, 11.71)	3.69 (2.27, 8.28) *^#$^
P		<0.001	<0.001	0.003	<0.001

*P < 0.0083 vs. the group <50. ^#^P < 0.0083 vs. the group 50–59. ^$^P < 0.0083 vs. the group 60–69. P values for pairwise comparisons were adjusted using the Bonferroni correction. The significance level for each comparison was set at 0.05/6 = 0.0083 to account for multiple comparisons among four age groups. PGI: Pepsinogen I; PGII: Pepsinogen II; PGR: PGI/PGII Ratio; G-17: Gastrin-17

### Correlations of serum PG and G-17 levels with age

**Fig 4:**
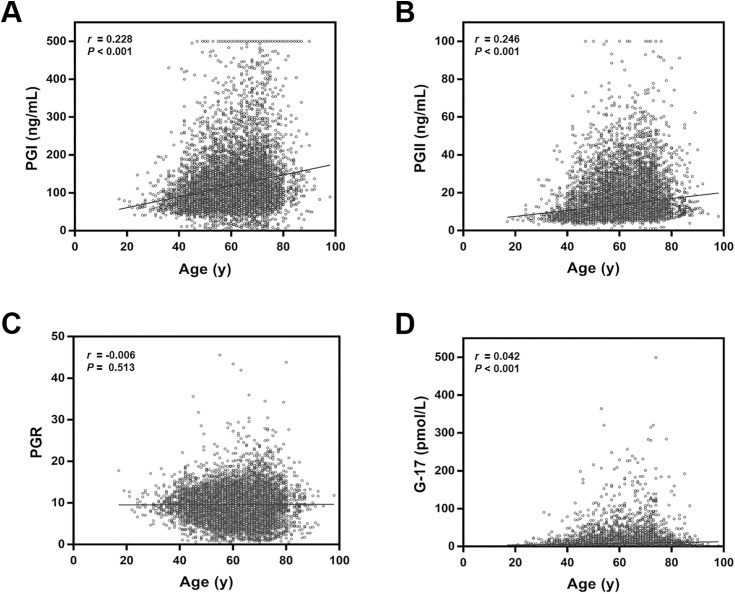
Correlations of serum PG and G-17 levels with age. (A) Correlation between serum PGI level and age; (B) Correlation between serum PGII level and age; (C) Correlation between PGR and age; (D) Correlation between serum G-17 level and age. PGI: Pepsinogen I; PGII: Pepsinogen II; PGR: PGI/PGII Ratio; G-17: Gastrin-17.

As shown in Table 2 and Fig 4, serum PGI, PGII, and G-17 levels were positively correlated with age, although the relationship was weak (r = 0.228, P < 0.001; r = 0.246, P < 0.001; r = 0.042, P < 0.001). The PGR was not correlated with age (r = −0.006, P = 0.513). Stratified analysis by HP status showed that in HP-negative individuals, age was positively correlated with PGI (r = 0.278, P < 0.001), PGII (r = 0.311, P < 0.001), and G-17 (r = 0.054, P < 0.001), but not with PGR (r = 0.010, P = 0.332). In HP-positive individuals, age was positively correlated with PGI (r = 0.072, P < 0.001) and PGII (r = 0.185, P < 0.001), negatively correlated with PGR (r = −0.170, P < 0.001), and showed no significant correlation with G-17 (r = 0.036, P = 0.071).

**Fig 5:**
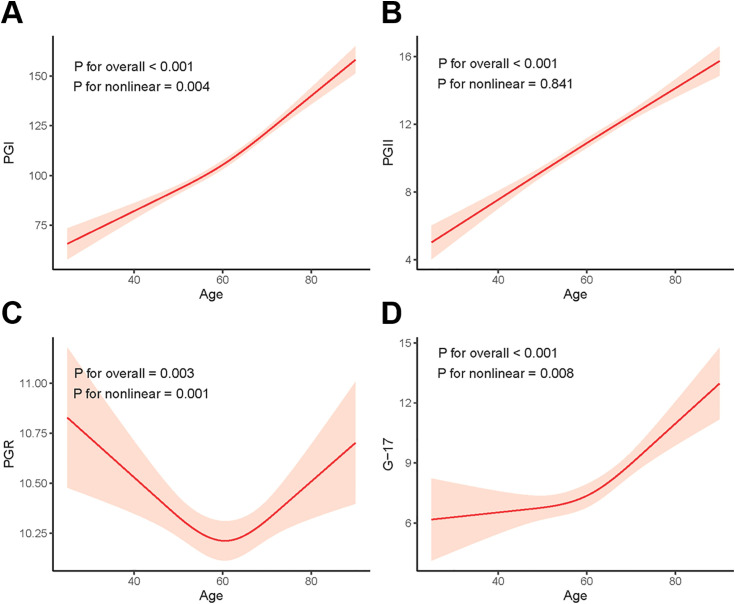
RCS analysis of the relationship between serum levels of PGI. **(A)**, PGII **(B)**, PGR **(C)**, and G-17 (D) with age, with covariates adjusted for sex and HP infection. The red line represents the adjusted association, with the 95% confidence interval shown in pink. RCS: Restricted cubic spline; PGI: Pepsinogen I; PGII: Pepsinogen II; PGR: PGI/PGII Ratio; G-17: Gastrin-17.

After adjusting for sex and HP infection covariates using an RCS, the analysis revealed a significant overall trend between PGI, PGII, PGR, and G-17 levels and age (P for overall < 0.001, P for overall < 0.001, P for overall = 0.003, and P for overall < 0.001, respectively). Specifically, we identified a nonlinear relationship between PGI, PGR, and G-17 levels and age (nonlinear P = 0.004, nonlinear P = 0.001, and nonlinear P = 0.008, respectively). In contrast, PGII demonstrated a linear correlation (nonlinear P = 0.841). These results are presented in Fig 5.

### Relationships of HP positive rate with age and sex

**Fig 6:**
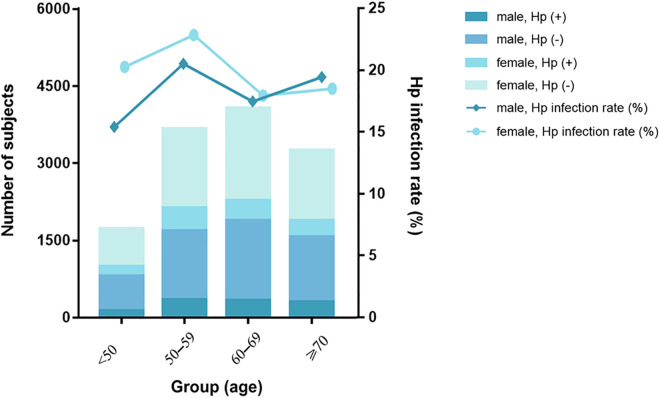
Associations of HP positive rate with sex and age.

Among 12,746 subjects who underwent the Anti-HP IgG Test to determine their HP infection status, we found 2,464 or 19.33%, were positive for HP. The HP positivity rates for males and females were 18.67% and 19.91%, respectively. However, the difference between the two groups was not statistically significant (χ² = 3.16, P = 0.075). Meanwhile, subjects in the 50–59 age group had the highest rate of HP infection at 21.82% (802/3676). Additionally, HP positivity significantly differed among subjects of various ages (χ² = 22.23, P < 0.001). Table 4 and Fig 6 list the relevant results.

**Table 4:** The distribution of HP infection positivity rates by sex and age groups.

Group	n	Patients HP Positive	HP Positive Rate (%)
Total	12746	2464	19.33
Male	5962	1113	18.67
Female	6784	1351	19.91
Patients by age (y)			
<50	1730	313	18.09
50–59	3676	802	21.82
60–69	4080	728	17.84
≥70	3260	621	19.05

### Associations of serum PG and G-17 levels with age and sex

**Fig 7:**
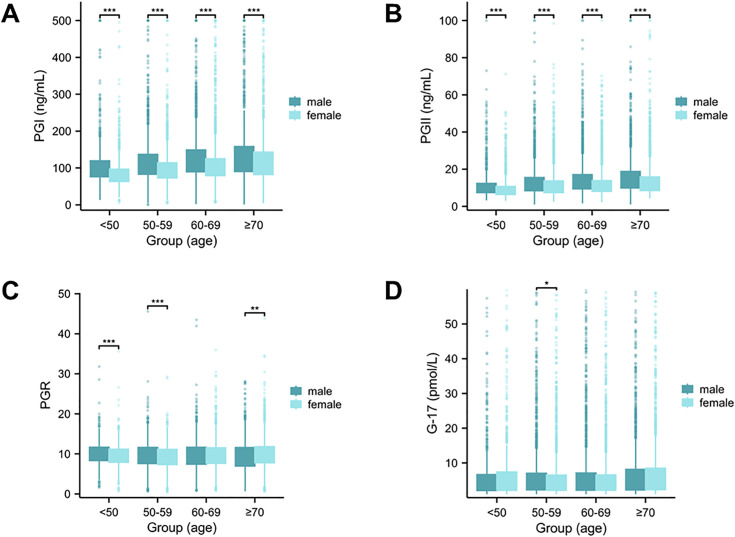
The association of serum PGs and G-17 with age and gender. The serum levels of PGI (A), PGII (B), PGR (C), and G-17 (D) among different gender and age groups. The serum PG and G-17 concentrations were determined by 12,746 cases, which were divided into 4 groups, including < 50 years-old group (n = 1,730), 50−59 years-old group (n = 3,676), 60−69 years-old group (n = 4,080), > 70years-old group (n = 3,260). Significant differences between the male group and female group (*P < 0.05, **P < 0.01, ***P < 0.01). PGI: Pepsinogen I; PGII: Pepsinogen II; PGR: PGI/PGII Ratio; G-17: Gastrin-17.

As shown in Fig 7A and 7B, serum levels of PGI and PGII were significantly higher in males compared to females across all age groups (P < 0.001, P < 0.001). A progressive increase in PGI and PGII levels with advancing age was observed in both genders. The PGR values showed significant differences between males and females in the < 50, 50–59, and ≥70 age groups (P < 0.001, P < 0.001, P < 0.01). No significant gender difference was observed in the 60–69 age group (Fig 7C). Furthermore, the G-17 levels were significantly higher in males than females in the 50–59 age group (P < 0.05), while no substantial sex differences were detected in other age groups (Fig 7D).

### Associations of Serum PG and G-17 Levels with Age and HP Positive Rate

**Fig 8:**
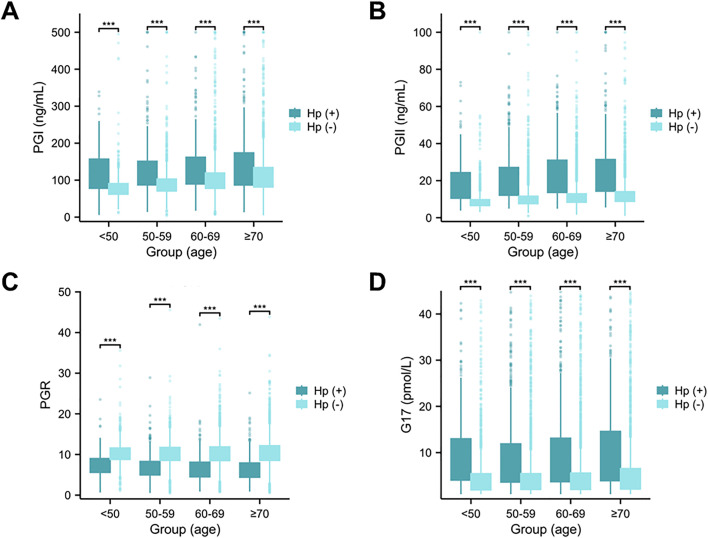
The association of serum PGs and G-17 with HP and gender. The serum levels of PGI (A), PGII (B), PGR (C), and G-17 (D) among different HP infection and age groups. The serum PG and G-17 concentrations were determined by 12,746 cases, which has been divided into 4 groups, including < 50 years-old group (n = 1,730), 50−59 years-old group (n = 3,676), 60−69 years-old group (n = 4,080), > 70years-old group (n = 3,260). Significant differences between the HP (+) group and HP (-) group (*P < 0.05, **P < 0.01, ***P < 0.01). PGI: Pepsinogen I; PGII: Pepsinogen II; PGR: PGI/PGII Ratio; G-17: Gastrin-17.

The serum levels of PGI, PGII, and G-17 were consistently higher in HP-positive individuals compared to HP-negative individuals across all age groups (P < 0.001, P < 0.001, P < 0.001) (Fig 8A, 8B, and 8D), while the PGR was lower than in HP-negative groups (P < 0.001) (Fig 8C).

### Detection of Abnormal Serum PG and G-17 in the subjects

In a cohort of 12,746 subjects, 2,639 cases (20.70%) had serum PGI levels outside the reference range (PGI < 70 ng/mL or PGI > 240 ng/mL); 4,168 cases (32.70%) exhibited PGII levels outside the reference range (PGII < 27 ng/mL); 327 cases (2.57%) had PGR levels below the reference range (PGR < 3); and 2,999 cases (23.53%) showed G-17 levels above the reference threshold (G-17 > 7.6 pmol/L). Additionally, 77 cases (0.60%) exhibited abnormal values across multiple markers: PGI < 70 ng/mL or PGI > 240 ng/mL, PGII > 13 ng/mL, PGR < 3, and G-17 > 7.6 pmol/L. Furthermore, the detection rate of abnormal serum PGI was significantly lower in males compared to females (17.28% vs. 23.72%, P < 0.001). Conversely, the detection rates for abnormal PGII and PGR were significantly higher in males than in females (37.99% vs. 28.05%, P < 0.001; 3.19% vs. 2.02%, P < 0.001, respectively). Relevant results are summarized in Table 5.

**Table 5:** Detection of Abnormal Serum PG and G-17.

Group	Case	PGI < 70 or PGI > 240 ng/mL Cases (%)	PGII > 13 ng/mL Cases (%)	PGR < 3 Cases (%)	G-17 > 7.6 pmol/L Cases (%)	PGI < 70 or PGI > 240 ng/mL + PGII > 13 ng/mL + PGR < 3 + G-17 > 7.6 pmol/L Cases (%)
male	5962	1030 (17.28)	2265 (37.99)	190 (3.19)	1426 (23.92)	41 (0.69)
female	6784	1609 (23.72)	1903 (28.05)	137 (2.02)	1573 (23.19)	36 (0.53)
χ²		80.20	142.45	17.30	0.94	1.30
P		<0.001	<0.001	<0.001	0.33	0.25

PGI: Pepsinogen I; PGII: Pepsinogen II; PGR: PGI/PGII Ratio; G-17: Gastrin-17.

### The relationship between HP infection and the detection rate of abnormal PG and G-17

Results showed that serum PGI, PGII, PGR, and G-17 thresholds were employed to assess the rates of abnormal values in both HP-positive and HP-negative groups, with the results presented in Table 6. The detection rates of abnormal PG and G-17 levels were significantly higher in the HP-positive group than in the HP-negative group (P < 0.01).

**Table 6:** Relationship between HP infection and detection rate of abnormal PG and G-17.

Group	Case	PGI < 70 ng/mL Cases (%)	PGI > 240 ng/mL Cases (%)	PGII > 13 ng/mL Cases (%)	PGR < 3 Cases (%)	G-17 > 7.6 pmol/L Cases (%)	PGI < 70 or PGI > 240 ng/mL + PGII > 13 ng/mL + PGR < 3 + G-17 > 7.6 pmol/L Cases (%)
HP+	2464	314 (12.74)	211 (8.56)	1840 (74.68)	156 (6.33)	1039 (42.17)	42 (1.70)
HP-	10282	1618 (15.74)	496 (4.82)	2328 (22.64)	171 (1.66)	1960 (19.06)	35 (0.34)
χ²		9.17	47.14	2445.40	173.27	589.72	61.60
P		0.002	<0.001	<0.001	<0.001	<0.001	<0.001

PGI: Pepsinogen I; PGII: Pepsinogen II; PGR: PGI/PGII Ratio; G-17: Gastrin-17.

## Discussion

This study assesses serum PG and G-17 levels in 12,746 asymptomatic subjects from Rizhao, Shandong, a coastal city in China, and analyzes the impact of HP infection on these biomarkers in this regional population. Our findings revealed that HP infection was positively correlated with PGI, PGII, and G-17, and negatively correlated with PGR. Moreover, the serum levels of PGI, PGII, and G-17 were consistently higher in HP-positive individuals compared to HP-negative individuals across all age groups, while the PGR was lower than in HP-negative groups, which is consistent with a previous study [22]. Notably, we observed that levels of PGII and G-17 were more than doubled in asymptomatic HP-infected individuals. The above results suggest that PGII and G-17 may be more sensitive than observed for PGI in HP-positive participants to gastric mucosal inflammation caused by HP. A possible explanation is that elevated gastrin levels stimulate G cells in the antrum, promoting the synthesis and secretion of PG in gastric cells, thus increasing both PGI and PGII levels [23]. However, as the corpus cells, including PGI-secreting cells, are lost, PGI levels decrease, while PGII remains elevated or stable [24]. Moreover, the increase in serum gastrin may be linked to elevated cytokine release near antral G cells [25,26], while in other instances, it may result from HP colonization of the gastric body and fundus, which increases local urea and ammonia levels. This, in turn, leads to reduced gastric acid secretion and a diminished inhibitory feedback mechanism on gastrin release [27,28]. The ROC curve analysis showed that the combined use of PGI, PGII, PGR, and G-17 demonstrated the highest AUC values, suggesting their potential in detecting HP infection (or past infection. However, while the colloidal gold method provides a rapid and cost-effective alternative, its diagnostic accuracy is lower than that of the urea breath test (UBT), which remains the gold standard due to its superior sensitivity and specificity [29]. Nonetheless, the colloidal gold method could be particularly useful in resource-limited and large-scale screening settings [30]. Additionally, this study revealed a significant difference in the detection rate of abnormal PG and G-17 between the HP-positive and HP-negative groups, which indicated that HP infection is one of the potential reasons for the observed abnormal PG and G-17. However, it is important to note that the study did not directly compare these biomarkers with confirmed gastric pathology, which remains a key limitation. Therefore, while these findings highlight the associations between serum PG and G-17 levels and HP infection, further research is required to better understand their role in reflecting gastric mucosal changes and their potential as biomarkers for HP infection.

In the present research, we found that the HP infection rate among asymptomatic individuals in China was 19.33%, lower than that previously reported at approximately 27.08% ~ 42.8% in China [31–33]. First, healthcare-seeking behavioral differences likely exist, as symptomatic individuals’ increased healthcare-seeking behavior leads to more frequent diagnosis. Additionally, surveillance bias may contribute to this pattern, as asymptomatic individuals are typically not targeted by active screening programs, resulting in a systematic underestimation of HP prevalence in this subgroup. Furthermore, the diagnosis of Helicobacter pylori in this study was based only on serological examination, which is not the gold standard for confirming HP infection, and this limitation may also contribute to the lower observed infection rate. This discrepancy, despite considering differences in participant selection (asymptomatic volunteers vs. the general population) and diagnostic methods, reflects the regional variability in HP infection rates. Potential factors contributing to these regional differences may include socioeconomic status, hygiene conditions, environmental factors, host genetics, dietary habits, and even differential sites of infection. Moreover, global trends indicate a significant decline in the prevalence of HP infection. A recent study by Li et al. [34] reported a reduction in the global estimated prevalence of HP from 58.2% (95% CI: 50.7–65.8) during 1980–1990 to 43.1% (95% CI: 40.3–45.9) during 2011–2022. We hypothesize that this decline may be closely linked to advancements in public health infrastructure, particularly improved sanitation standards, expanded accessibility to medical services, and the extensive utilization of antibiotic therapies. Given the ongoing process of urbanization in China, the prevalence of HP infection may continue to decline in the future.

Our study suggested that sex affected the PG and G-17. Our study showed that males exhibited significantly higher levels of both PGI and PGII than females, irrespective of HP infection status, which is consistent with previous studies [35,36]. Importantly, our stratified analysis by HP infection status (Fig 2, Table 2) reveals that the sex effect on PG levels is dependent on infection status. In HP-negative individuals, males had higher PGI and PGR, aligning with known gender differences in gastric secretion. In HP-positive individuals, however, the male predominance in PGI disappeared, and PGR was inversely associated with male sex, suggesting that HP infection may alter sex-specific gastric homeostasis. Regarding G-17, the influence of gender on serum levels remains controversial [33,37,38]. G-17 is an unstable indicator that is affected by many factors, likely due to regional and population-specific variations. Notably, an exception was observed in the 50−59 age group, where G-17 levels were higher in males than in females. This finding aligns with the results reported by Liu et al. [38], who suggested that such differences may be related to the changes in hormones that occur in women during menopause. Given the influence of gender on serum levels of PG and G-17, our findings warrant clinical reconsideration. Gender-specific reference intervals should be stratified by HP infection status, particularly for PGI and PGR, where infection appears to reverse the typical sex effects. Moreover, HP-positive males—who exhibit paradoxical PGR suppression—represent a high-risk phenotype and should be closely monitored.

In addition, our results showed that age was a factor influencing serum PG and G-17 levels. Correlation analysis revealed weak positive correlations between serum PGI, PGII, G-17 levels, and age in the overall cohort. In HP-negative individuals, these nonlinear associations were consistent with known physiological changes in mucosal aging. However, in HP-positive individuals, the relationship between age and biomarkers was altered, suggesting that HP infection may accelerate or modulate age-related changes in gastric function. Notably, although Spearman analysis did not show a significant linear correlation between PGR and age, age-group comparisons revealed significant inter-group differences for PGR. Furthermore, after adjusting for sex and HP infection using the RCS model, a marked nonlinear relationship between PGR and age was identified. However, this contrasts with the linear trend reported by Liu et al. [39] and may stem from several differences: (1) Traditional linear models, such as Spearman correlation, may fail to capture the complex dynamic relationships between age and biomarkers. For example, PGI and G-17 levels may be regulated by different mechanisms in various age groups (e.g., active mucosal metabolism in young adulthood vs. atrophic changes in older age), whereas the RCS model, by flexibly fitting nonlinear trends, more accurately captures these multi-stage effects; (2) Our study focused primarily on middle-aged and elderly individuals (50−80 years), whereas Liu et al. included a broader age range (20−80 years). In our study, the limited range of PGR variations and substantial overlap in the interquartile ranges across age groups may have masked early-life changes in PGR; (3) The unbalanced distribution of samples in our study may have affected the statistical power to detect trends. To better understand the relationship between age and biomarkers such as PGR, PGI, PGII, and G-17, future studies should consider a broader and more balanced age and sex distribution and increase sample size to ensure the full spectrum of biomarker changes across the lifespan is captured.

Several limitations of our study should be acknowledged. First, the asymptomatic participants included in this study did not undergo gastroscopy or pathological examination, meaning that some asymptomatic patients with other underlying gastric conditions could not be excluded. As a result, the findings may not accurately reflect the true gastric status. Second, this study assessed the prevalence of HP infection in a cross-sectional population using only the colloidal gold method. However, the results may underestimate the true infection rate due to the method’s limited sensitivity and specificity compared to gold standards like the UBT. Additionally, the lack of consideration for high negative titers may further affect the accuracy of identifying true negative cases, leading to potential underestimation. Furthermore, the lack of stratification by HP infection status represents a significant limitation. Due to the use of serum IgG antibodies as a marker for HP infection, we were unable to differentiate between participants who were never infected, currently infected, or previously infected, as antibodies persist after eradication. HP infection rates vary across different age and sex groups, and without stratifying participants based on infection status, it remains unclear whether the observed changes in serum markers, such as gastrin, are attributable to physiological aging or the differing prevalence of HP infection across these groups. Future studies should address this limitation by incorporating more reliable diagnostic methods, such as UBT or stool antigen testing, to enable accurate stratification of participants based on HP infection status and confirm these findings.

In conclusion, our study demonstrates that serum PG and G-17 levels are associated with HP infection, sex, and age. These findings provide region-specific insights into the relationships between these biomarkers and HP infection, highlighting the importance of considering HP infection status, sex, and age in future research and clinical practice for a better understanding of gastric disease pathophysiology.

## Supporting information

S1 Table Diagnostic Performance of Various Marker Combinations. (DOCX)

S1 Data Data. (XLSX)
