# Supplementary material for: Associations of serum pepsinogen and Gastrin-17 with Helicobacter pylori infection, sex, and age, in an asymptomatic coastal population: A cross-sectional study in Rizhao, China
Source: PLoS One. 2025 Nov 4;20(11):e0335228. doi: 10.1371/journal.pone.0335228 (PMC12585035; doi:10.1371/journal.pone.0335228)
Supplement: S1 Table — (DOCX) [file pone.0335228.s001.docx]

**Supplementary Table 1 Diagnostic Performance of Various Marker Combinations**

|  | AUC | cut-off | Sensitivity (%) | Specificity (%) | Accuracy (%) | PPV (%) | NPV (%) | 95%CI |
| --- | --- | --- | --- | --- | --- | --- | --- | --- |
| PGI | 0.605 | 115.500 | 51.50 | 67.16 | 64.13 | 27.31 | 85.25 | 0.592 – 0.618 |
| PGII | 0.805 | 13.450 | 72.93 | 79.42 | 78.17 | 45.92 | 92.45 | 0.795 – 0.816 |
| PGR | 0.820 | 7.745 | 70.09 | 84.05 | 81.35 | 51.29 | 92.14 | 0.811 – 0.830 |
| G-17 | 0.709 | 3.685 | 75.93 | 59.08 | 62.34 | 30.78 | 91.11 | 0.698 – 0.720 |
| PGI+PGII | 0.841 | - | 74.59 | 85.51 | 83.40 | 55.23 | 93.35 | 0.832 – 0.851 |
| PGI+PGR | 0.837 | - | 75.37 | 83.58 | 81.99 | 52.38 | 93.40 | 0.828 – 0.847 |
| PGI+G-17 | 0.819 | - | 70.01 | 83.68 | 81.04 | 50.69 | 92.09 | 0.809 – 0.829 |
| PGII+PGR | 0.839 | - | 75.65 | 83.11 | 81.67 | 51.76 | 93.44 | 0.829 – 0.848 |
| PGII+G-17 | 0.805 | - | 73.54 | 78.70 | 77.70 | 45.28 | 92.54 | 0.795 – 0.815 |
| PGR+G-17 | 0.819 | - | 70.01 | 83.68 | 81.04 | 50.69 | 92.09 | 0.809 – 0.829 |
| PGI+PGII+PGR | 0.838 | - | 75.12 | 83.79 | 82.11 | 52.62 | 93.36 | 0.828 – 0.847 |
| PGI+PGII+G-17 | 0.841 | - | 74.35 | 85.58 | 83.41 | 55.26 | 93.30 | 0.832 – 0.851 |
| PGI+PGR+G-17 | 0.838 | - | 74.68 | 84.15 | 82.32 | 53.03 | 93.27 | 0.829 – 0.847 |
| PGII+PGR+G-17 | 0.839 | - | 74.47 | 84.00 | 82.16 | 52.73 | 93.21 | 0.829 – 0.848 |
| PGI+PGII+PGR+G-17 | 0.838 | - | 74.76 | 84.09 | 82.29 | 52.96 | 93.29 | 0.829 – 0.848 |

HP: *Helicobacter pylori*; PGI: Pepsinogen I; PGII: Pepsinogen II; PGR: PGI/PGII Ratio; G-17: Gastrin-17; PPV: positive predictive value; NPV: negative predictive value.
